# Electrochemical Characterization of *Escherichia coli* Adaptive Response Protein AidB

**DOI:** 10.3390/ijms131216899

**Published:** 2012-12-11

**Authors:** Michael J. Hamill, Marco Jost, Cintyu Wong, Nicholas C. Bene, Catherine L. Drennan, Sean J. Elliott

**Affiliations:** 1 Department of Chemistry, Boston University, 590 Commonwealth Ave., Boston, MA 02215, USA; E-Mails: mike.j.hamill@gmail.com (M.J.H.); nchangbene@gmail.com (N.C.B.); 2 Department of Chemistry, Massachusetts Institute of Technology, 77 Massachusetts Ave., Cambridge, MA 02139, USA; E-Mails: mjost@mit.edu (M.J.); cdrennan@mit.edu (C.L.D.); 3 Department of Biology, Massachusetts Institute of Technology, Cambridge, MA 02139, USA; 4 Howard Hughes Medical Institute, Massachusetts Institute of Technology, Cambridge, MA 02139, USA; 5 Center for Environmental Health, Massachusetts Institute of Technology, Cambridge, MA 02139, USA

**Keywords:** adaptive response, DNA repair, protein electrochemistry, acyl-coenzyme A dehydrogenase, flavin cofactor, reduction potential

## Abstract

When exposed to known DNA-damaging alkylating agents, *Escherichia coli* cells increase production of four DNA repair enzymes: Ada, AlkA, AlkB, and AidB. The role of three enzymes (Ada, AlkA, and AlkB) in repairing DNA lesions has been well characterized, while the function of AidB is poorly understood. AidB has a distinct cofactor that is potentially related to the elusive role of AidB in adaptive response: a redox active flavin adenine dinucleotide (FAD). In this study, we report the thermodynamic redox properties of the AidB flavin for the first time, both for free protein and in the presence of potential substrates. We find that the midpoint reduction potential of the AidB flavin is within a biologically relevant window for redox chemistry at −181 mV, that AidB significantly stabilizes the flavin semiquinone, and that small molecule binding perturbs the observed reduction potential. Our electrochemical results combined with structural analysis allow for fresh comparisons between AidB and the homologous acyl-coenzyme A dehydrogenase (ACAD) family of enzymes. AidB exhibits several discrepancies from ACADs that suggest a novel catalytic mechanism distinct from that of the ACAD family enzymes.

## 1. Introduction

*Escherichia coli* adaptive response is a process initiated by the exposure of cells to low doses of DNA alkylating agents, which have mutagenic and cytotoxic effects on cells [1–3]. The adaptive response process refers to the up-regulation of four proteins, Ada, AlkA, AlkB, and AidB, that reduce the effects of alkylation on DNA [3,4]. Out of these four proteins, three have known roles in the adaptive response as enzymes that repair DNA lesions via a variety of mechanisms: Ada as a methyltransferase [5–8], AlkA as a glycosylase [9,10], and AlkB as a dioxygenase [11–15]. A complete understanding of the fourth protein, AidB, remains elusive.

**Scheme 1:**
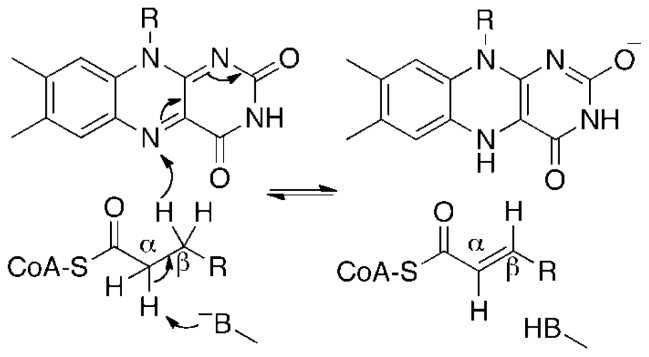
Generic mechanism for dehydrogenation of a substrate by an acyl-coenzyme A dehydrogenase.

By sequence homology, AidB is similar to members of the acyl-coenzyme A dehydrogenase (ACAD) enzyme family [16], which contain a redox active flavin adenine dinucleotide (FAD) cofactor that catalyzes the α,β-dehydrogenation of acyl-coenzyme A (CoA) substrates (Scheme 1). AidB similarly contains a FAD cofactor and displays isovaleryl-coenzyme A dehydrogenase (IVD) activity [16,17]. This activity, however, is 1000-fold lower than that of known ACADs and therefore probably not biologically relevant to AidB function [16,17]. The observation of bound FAD, combined with evidence for DNA binding, led to the hypothesis that a flavin-dependent dehydrogenation reaction similar to reactions catalyzed by ACADs could be used to repair DNA lesions [17]. This model was superseded when the first crystal structure of AidB provided evidence that a direct reaction between the flavin and DNA is unlikely due to spatial constraints. Instead, based on crystal packing data it was proposed that AidB sheaths DNA by forming higher-ordered structures of tetramers, generating pores for DNA binding and resulting in steric protection [18]. In this model, flavin has a role protecting DNA via an unknown redox mechanism that deactivates harmful alkylating agents [18]. Significant *in vivo* evidence for AidB protection of DNA against the alkylating agents *N*-methyl-*N*′-nitro-*N*-nitrosoguanidine (MNNG), *N*-ethyl-*N*′-nitro-*N*-nitrosoguanidine, and methyl methanesulfonate (MMS) has recently been reported [19], although it has also been shown AidB does not directly react with MNNG [20]. Surprisingly, the DNA-binding domain of AidB is not required for protection activity, making a steric protection mechanism unlikely [19,21]. Rather, it is suggested that AidB uses its DNA-binding domain to preferentially localize to regions of DNA, so-called UP elements, found upstream of ribosomal RNA genes, tRNA genes, and DNA repair genes as a means to localize detoxification activity to these crucial genes [19].

A mechanism by which AidB prevents DNA damage by detoxifying alkylating agents has not been proposed. Since no activity besides that with isovaleryl-coenzyme A (IVCoA) has been reported, the role of bound flavin in AidB’s detoxification mechanism is unclear. In our previous work we demonstrated that flavin clearly plays a structural role in AidB by inducing formation of a tetramer [21]. This observation motivates a lingering question: does the AidB flavin also play a role in redox chemistry? This possibility was most recently studied by Mulrooney *et al*. who observed the reduction of AidB by *E. coli* flavodoxin (FldA), but at slow electron transfer rates that suggest it is not physiologically relevant [20].

To determine the possibility of an enzymatic flavin role, we extensively characterized the redox properties of AidB to place AidB in the context of known, specific flavin chemistries. Furthermore, we investigated the option of redox modulations caused by potential AidB substrates such as IVCoA or DNA, providing a comparison between AidB and the ACAD family of enzymes that have well-characterized and distinctive redox properties. Here we show for the first time that AidB is capable of mediating redox chemistries within a physiologically relevant window of reduction potential. In addition, the ability of AidB to generate a significant amount of flavin semiquinone suggests a role for AidB as a redox enzyme, but with chemistries distinct from that of the ACAD family.

## 2. Results

### 2.1. Determination of Reduction Potentials for AidB

**Figure 1:**
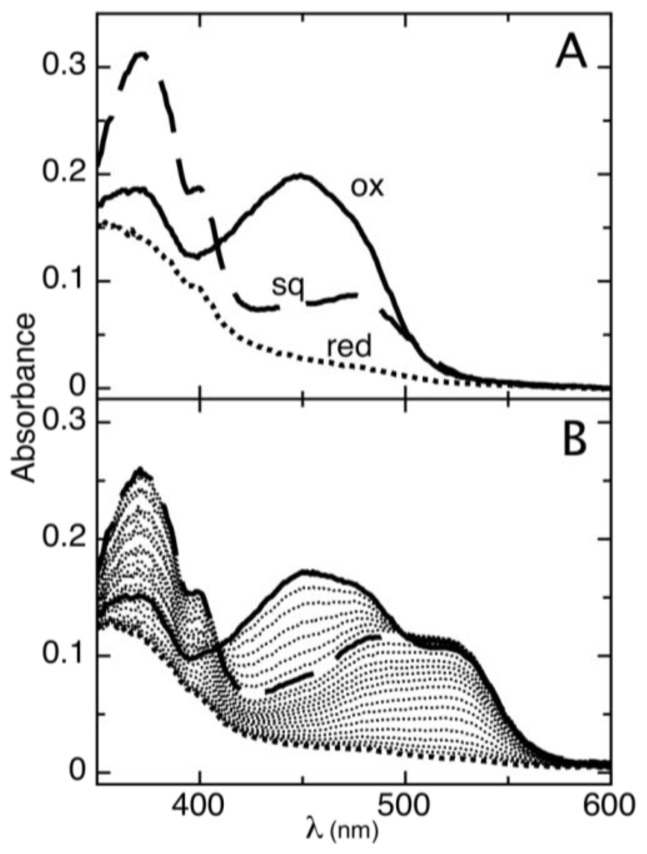
Complete anaerobic reduction of AidB using the xanthine/xanthine oxidase method. (**A**) Representative spectra of the three redox states observed for AidB: oxidized (solid line), anionic semiquinone (dashed line), and reduced (dotted line); (**B**) Reduction of AidB in the presence of the redox dye PS (maximum absorbance at 520 nm), showing spectra collected every ~5 min.

**Figure 2:**
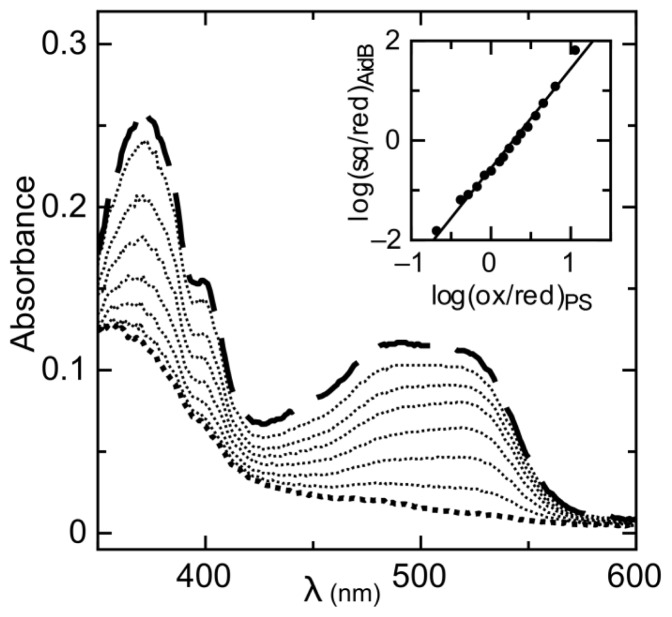
Simultaneous reduction of the AidB anionic semiquinone and PS. The visible absorbance spectra for the anionic semiquinone (monitored at 406 nm) and PS (monitored at 555 nm) decrease during their reduction. Inset: Plot of log [ox]/[red] for PS *vs*. log [sq]/[red] for AidB, used to calculate the midpoint potential with respect to the reference value of PS (−283 mV, pH 8).

The reduction of AidB occurs in two resolvable phases (Figure 1). The first phase is the reduction of oxidized flavin (ox) to the distinct anionic semiquinone (sq) that has a characteristic absorbance maximum at lower wavelengths [22]. The absorbance maximum for the AidB anionic semiquinone is 365 nm (in agreement with 370 nm typically observed for anionic semiquinones in other flavoenzymes [23]), with an isosbestic point at 409 nm observed during the transition from the oxidized to the semiquinone species (Figure 1A). The second phase of reduction is the transition from the anionic semiquinone to fully reduced flavin (red). This phase is observed simultaneously with the reduction of phenosafranine (PS), indicative of similar reduction potentials (Figure 1B). Absorbance of AidB semiquinone at 406 nm during the second phase of reduction was used to monitor the transition from oxidized to reduced, and these data were plotted *versus* the reduction of PS as described in the experimental procedures. We determine a reduction potential of *E_m_^sq/red^* = −258 ± 6 mV for the sq/red couple, with a log plot slope of 1.96, indicative of comparing two-electron and one-electron processes for PS and AidB, respectively (Figure 2).

**Figure 3:**
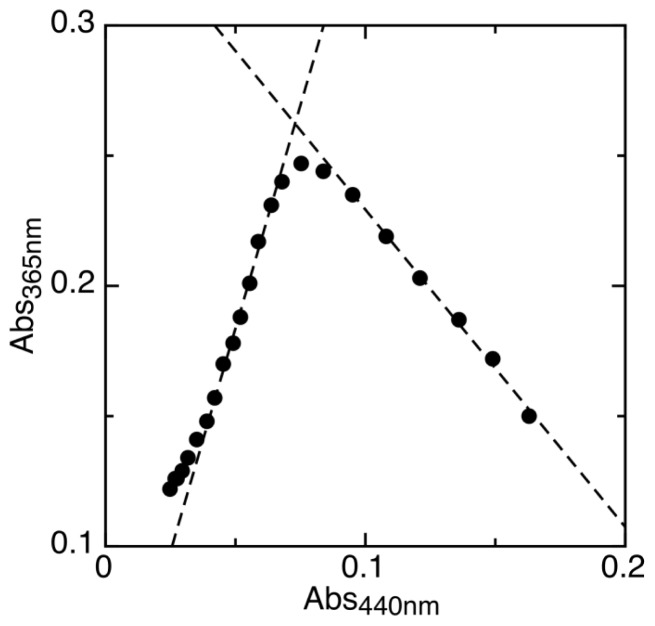
Maximum proportion of semiquinone formed by AidB during reduction is determined by plotting absorbance values at 365 nm *vs*. 440 nm. The linear fits, shown as dashed lines, are used to determine the theoretical absorbance of complete semiquinone formation.

To analyze the first step of reduction, we measured the maximum amount of semiquinone intermediate formed during the experiment (Figure 3). This fraction corresponds to the semiquinone formation constant (*K_sq_*, Equation 1) according to Equation 2 as derived by Clark [24]. *K_sq_* reflects the difference in midpoint potentials of the two single-electron redox transitions as shown in Equation 3.

(1) $$K_{sq}=\frac{{\left[\text{sq}\right]}^{2}}{\left[\text{ox}][\text{red}\right]}$$

(2) $${\left(\frac{\left[\text{sq}\right]}{\left[\text{AidB}\right]}\right)}_{\text{max}}=\frac{\sqrt{K_{sq}}}{2+\sqrt{K_{sq}}}$$

(3) $${E_{m}}^{ox/sq}-{E_{m}}^{sq/red}=2.303(RT/F)\text{log}\;K_{sq}$$

Where *K_sq_* is the semiquinone formation constant, ([sq]/[AidB])*_max_* is the maximum proportion of semiquinone formed, and *E_m_^ox/sq^* and *E_m_^sq/red^* are the one-electron reduction potentials of flavin. Since *E_m_^sq/red^* has already been measured (−258 mV), the oxidized to semiquinone reduction potential was calculated, *E_m_^ox/sq^* = −103 ± 10 mV. The midpoint potential for the two-electron reduction of AidB (*E_m_^ox/red^*) was also calculated from the average of *E_m_^ox/sq^* and *E_m_^sq/red^*, *E_m_^ox/red^* = −181 mV.

### 2.2. Effect of IVCoA on Reduction Potential

**Figure 4:**
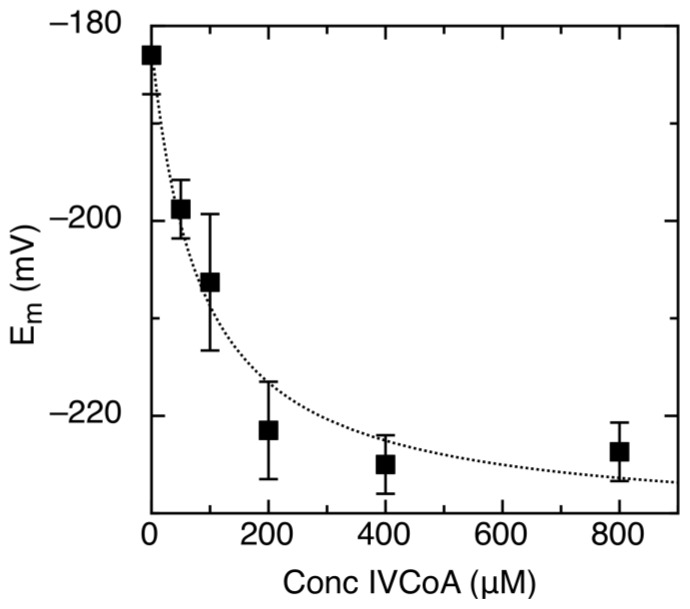
Midpoint reduction potential (*E_m_*) of AidB lowers as a function of isovaleryl-CoA (IVCoA) concentration. The dotted line is the fit to equation 4 to determine *K_d_^app^*.

To better understand possible AidB dehydrogenase activity, we studied the redox properties as a function of IVCoA concentration. As expected, anaerobic incubation of IVCoA with AidB did not result in any reduction or alteration of the FAD absorption spectrum [17]. Reduction in the presence of saturating IVCoA concentrations significantly shifted both potentials in the negative direction to *E_m_^ox/sq^* = −148 ± 10 mV and *E_m_^sq/red^* = −297 mV ± 5 mV, a −42 mV change in *E_m_*. The amount of semiquinone formed was unaffected by the presence of IVCoA. The dependence of *E_m_* on [IVCoA] was used to determine the apparent dissociation constant (*K_d_^app^*, Figure 4). The shift in reduction potential was fit to a simple two-state binding model (Equation 4) for determining the dissociation constant.

(4) $$E_{m}=({E_{m}}^{\text{max}}-{E_{m}}^{0})\left(\frac{\left[\text{IVCoA}\right]}{{K_{d}}^{app}+[\text{IVCoA}]}\right)+{E_{m}}^{0}$$

Where *E_m_* is the midpoint potential at a given IVCoA concentration, *E_m_*^0^ is the midpoint potential without IVCoA, *E_m_*^max^ is the reduction potential at saturating concentrations of IVCoA, and *K_d_^app^* is the apparent dissociation constant. The determined *K_d_^app^* = 87 ± 27 μM represents binding of IVCoA to oxidized, semiquinone, and reduced forms of the enzyme. Binding of IVCoA causes a negative shift in reduction potential, which indicates a preference for and stabilization of oxidized AidB.

### 2.3. Ionic Strength Dependent Reduction Potentials

**Figure 5:**
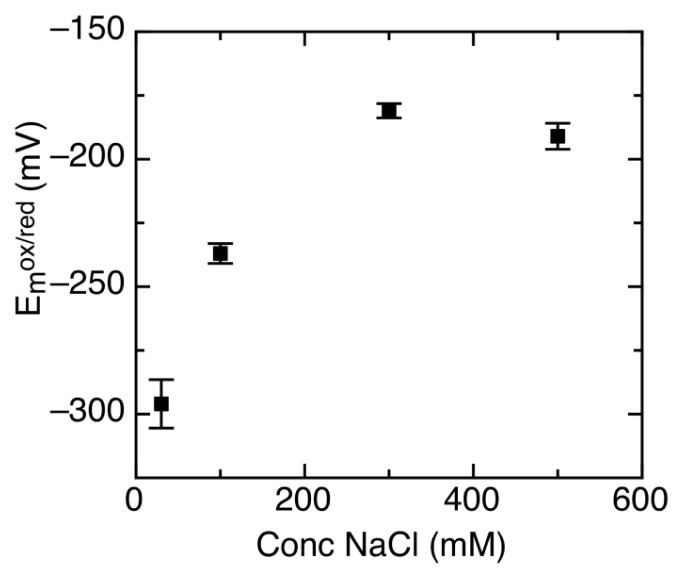
Midpoint reduction potential (*E_m_*) of AidB at various NaCl concentrations.

It has been shown that AidB binds DNA using its *C*-terminal positively charged domain [18]. Due to the electrostatic nature of this interaction, it is highly dependent on ionic strength [17,18,21]. To measure changes in redox properties when AidB is exposed to DNA, the reduction potential had to first be characterized without DNA in the presence of 100 mM NaCl, which is a concentration of NaCl where AidB is known to have a high affinity for DNA [17,21]. Compared to 300 mM NaCl in earlier measurements, lowering the NaCl concentration to 100 mM shifts the reduction potential to significantly more negative values. At 100 mM NaCl, reduction potentials are: *E_m_^ox/sq^* = −160 ± 6 mV and *E_m_^sq/red^* = −315 ± 4 mV, with no change in the amount of semiquinone formed. Further studies at a broader NaCl range showed that below 300 mM NaCl, the AidB reduction potential depends strongly on NaCl concentration (Figure 5).

### 2.4. Effect of DNA on Reduction Potential

After determining the effects of ionic strength on AidB redox properties, we could determine the impact of DNA on the observed reduction potentials. AidB binds several forms of DNA, ranging from small oligomers (25 bp) [18] to entire plasmids such as pUC19 [17]. To determine if DNA binding regulates AidB’s redox properties, we measured reduction potentials under saturating DNA concentrations. Two forms of DNA were used for binding during reductions: pUC19 and a double stranded 28 bp oligomer containing the UP element in addition to the −35 box of the rrnB P1 promoter (5′-GAAAATTATTTTAAATTTCCTCTTGTCA-3me;). The sequence for the 28-mer was chosen because AidB offers above average protection from damage to this DNA segment [19]. Measurements with pUC19 included 500 ng/mL DNA (322 bp per AidB tetramer), and measurements with the 28-mer included 214 ng/mL DNA (154 bp per AidB tetramer). Reduction potential measurements were completed in lowered ionic strength solutions (100 mM NaCl) as described above. Both plasmid and the 28-mer resulted in a positive shift of the reduction potentials of AidB. Addition of pUC19 to the reduction of AidB at 100 mM NaCl shifted the potentials positively *E_m_^ox/sq^* = −121 ± 11 mV, *E_m_^sq/red^* = −274 ± 5 mV, with no change in the yield of the AidB semiquinone. Similarly, the 28-mer shifted the potentials positively *E_m_^ox/sq^* = −129 ± 11 mV and *E_m_^sq/red^*= −282 ± 5 mV, also with no change in the amount of semiquinone.

### 2.5. AidB-ETF Docking Model

**Figure 6:**
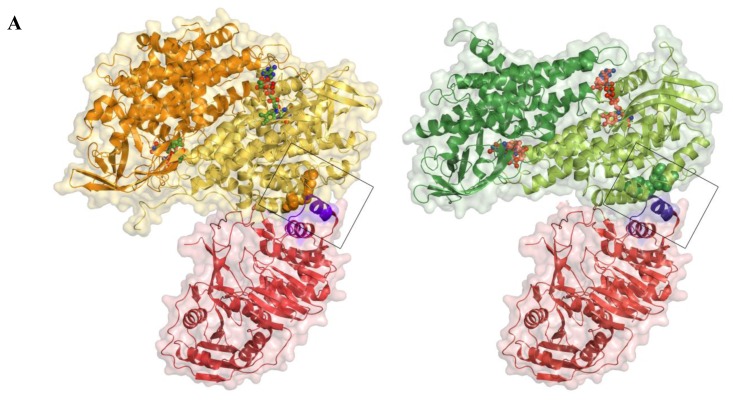

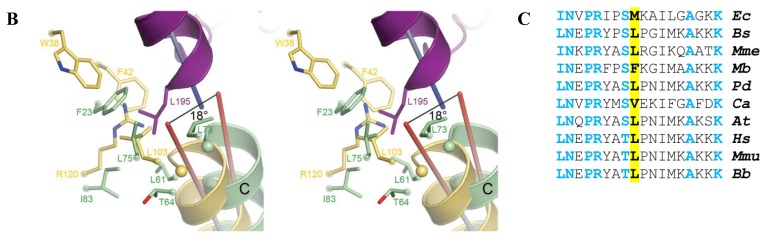
Model of the docking between AidB and the human ETF. (**A**) Side-by-side views of the AidB-ETF docking model (left) and the MCAD-ETF complex structure (right), shown in the same orientation. For the docking, the crystal structure of AidB (PDB ID: 3U33 [21]) was superimposed onto the MCAD-ETF complex structure (PDB ID: 1T9G [29]). All structures are shown in ribbon representation, with the ETF in red, AidB protomers in yellow and orange, and MCAD protomers in dark and light green. Only one dimer is shown both for the AidB tetramer and the MCAD tetramer, the second dimer is omitted for clarity in both cases. Transparent surfaces are shown around all proteins in red (ETF), yellow (AidB) and green (MCAD). Bound FAD-cofactors of AidB and MCAD are shown in ball-and-stick representation with carbon atoms in light green and orange, respectively. The ETF recognition loop is shown in purple and interacting hydrophobic residues of AidB and MCAD are shown as orange and green spheres, respectively; (**B**) Wall-eyed stereo view of the ETF recognition loop interactions. The docking was generated in the same fashion as in (A). The recognition loop is shown in purple ribbons, with Leu195 shown as sticks. MCAD residues from the MCAD-ETF complex structure that are interacting with the recognition loop are shown with pale green carbons. AidB residues near the putative location of ETF Leu195 are shown with yellow carbons. The axes of the recognition loop helix, MCAD helix C, and the corresponding AidB helix are shown and colored by dipole moment from blue (positive) to red (negative); (**C**) Multiple sequence alignment of ETFs from different organisms. The residue that inserts into the hydrophobic pocket of partner proteins is highlighted in yellow. Other conserved residues are shown in blue. *Ec*, *E. coli* (protein YdiQ); *Bs*, *Bacillus subtillis; Mme*, *Methylophilus methylotrophus; Mb*, *Mycobacterium bovus; Pd*, *Paracoccus denitrificans; Ca*, *Clostridium acetobutylicum; At*, *Arabidopsis thaliana; Hs*, *Homo sapiens; Mmu*, *Mus musculus; Bb*, *Bos bovus*.

If an ideal substrate for the AidB reductive half reaction exists, a mechanism would need to be in place to allow for reoxidation of the flavin cofactor. For ACADs such as medium-chain acyl-coenzyme A dehydrogenase (MCAD) and IVD, an electron transfer flavoprotein (ETF) accepts the electrons from the reduced flavin of the ACAD and further transfers them to the respiratory chain [25]. Structures of ETFs from *Paracoccus denitrificans*[26] and human [27] are known, and the determinants of binding to ACADs and other partner proteins are well-characterized on the basis of crystal structures of the human MCAD-ETF complex and a complex of *Methylophilus methylotrophus* ETF with trimethylamine dehydrogenase (TMADH) [28,29]. To examine whether AidB could interact with an ETF, we generated models of a complex between AidB and the human ETF using the known crystal structure of the MCAD-ETF complex (Figure 6).

When considering docking of the ETF to an AidB tetramer, which is the predominant state of AidB in solution [21], one dimeric unit of AidB is unfavorably close to the human ETF FAD-binding domain (not shown). However, the implications of this clash are difficult to assess, as the ETF FAD-binding domain is thought to be flexible both when the ETF is free in solution and in complex with its partner proteins [25,29–31]. In terms of docking with an AidB dimer, here the overall shape complementarity is favorable and similar to the human MCAD-ETF complex (Figure 6A) and the bacterial TMADH-ETF complex (not shown). Critical determinants for efficient electron transfer, however, are missing. In particular, both MCAD and TMADH present a hydrophobic pocket to the ETF recognition loop (residues 191–200, human numbering), which inserts a conserved hydrophobic residue (Leu195, human numbering) into this pocket (Figures 6A, 6B) [28,29]. Mutation of Leu195 or proteolytic removal of the recognition loop abolishes electron transfer between the ETF and its partner proteins, and mutational studies have furthermore demonstrated that the size of the hydrophobic pocket is also critical for efficient electron transfer [28,29]. Notably, even though MCAD and TMADH bind to the ETF in the same manner, they share no structural homology with each other, suggesting that the ETF uses the same binding mode for a range of structurally dissimilar partner proteins. While AidB contains a number of hydrophobic residues in the corresponding region, including Trp38, Phe42, and Leu103, the guanidinium moiety of AidB Arg120 would be pointed directly at the hydrophobic residue inserted by the ETF, providing an unfavorable interaction (Figure 6B). Arg120 is stabilized in this position by interaction with AidB Asp39 (not shown). Furthermore, helix C in AidB (residues 88–98) is tilted by 18° and appears unable to achieve the ideal alignment of the corresponding helix in MCAD (residues 50–60) with the ETF recognition loop helix, which is thought to stabilize the MCAD-ETF complex [28,29] (Figure 6B). Although the AidB helix could adopt a more favorable position upon binding of the ETF, no change is observed for MCAD upon ETF binding [29,32].

The docking was carried out with the human ETF, but the critical residues are conserved in the *M. methylotrophus* ETF and the *E. coli* ETF (Figure 6C). In particular, Leu195 is Met, which can likely insert into the hydrophobic pocket in the same mode, and the hydrophobic Ile is found in position 191. In addition, I/L186, N187, P189, R190, and S/T194, which are thought to be important to stabilize the recognition loop structure, are conserved. A homology model of the *E. coli* ETF furthermore indicates that the *E. coli* ETF likely has the same conserved structure (not shown).

## 3. Discussion

In this work, we have characterized the thermodynamic redox properties of AidB for the first time. We report the two one-electron reduction potentials for flavin bound to AidB, oxidized to semiquinone and semiquinone to reduced. Although this is the first characterization of AidB reduction potentials, the closely related ACAD family has been extensively studied [33]. A comparison of the AidB electrochemical properties reported here and those of homologous ACAD enzymes reveals both intriguing similarities and differences.

The two-electron midpoint potential we have measured for AidB (*E_m_^ox/red^* = −181 mV) is at the lower end of the −60 to −180 mV range reported for the two-electron reduction of ACAD enzymes at neutral pH values [33–38]. We also find that AidB stabilizes a large amount (92%) of the anionic semiquinone intermediate, a result that agrees with the reported single electron reduction of AidB using photoreduction methods [17]. In contrast, ACADs typically yield 0%–20% semiquinone. Stabilization of 0%–20% semiquinone intermediate indicates that the second one-electron reduction of the ACAD flavin is more thermodynamically favorable than the first, as denoted by a higher reduction potential. This coupling is essential because it allows for the two-electron transfer required during the dehydrogenation mechanism of ACADs. Since AidB stabilizes 92% of the anionic semiquinone, the second one-electron reduction step is less thermodynamically favorable by −155 mV. Uncoupling of the two one-electron reduction steps to this extent significantly hinders the ability for AidB to extract two electrons as needed for ACAD activity. Thus, these data indicate that AidB could be involved in a different type of reaction, possibly involving one-electron transfers.

In general, the reduction potentials of ACADs are more negative than the reduction potentials of their acyl-CoA substrates, which themselves have reduction potentials of roughly −40 mV [34]. To generate a thermodynamically favorable electron transfer from acyl-CoA to flavin, a large positive shift in the reduction potential of the bound flavin occurs when ACAD enzymes bind their biological substrates. This trend has been demonstrated in several ACADs from various sources, including short-chain acyl-CoA dehydrogenase (SCAD) from *Megasphaera elsdenii* (*Me*SCAD) [35–37], MCAD from pig kidney (*p*MCAD) [34,38], glutaryl-CoA dehydrogenase from *Paracoccus denitrificans* (*Pd*GCD) [39], and human SCAD (*h*SCAD) [40] (Table 1). This substrate-dependent redox modulation, which facilitates dehydrogenation of the appropriate substrate, is a distinctive redox property of ACADs. Such modulation could be an important feature retained in AidB that is necessary for catalytic activity.

We therefore measured the AidB reduction potentials in the presence of the putative substrates IVCoA and DNA. Notably, IVCoA induces a decrease in the AidB flavin reduction potentials, which expands the overall difference in reduction potential between the acyl-CoA and the flavin. A similar phenomenon of decreasing flavin reduction potentials has been observed for ACADs in the presence of non-natural substrates (Table 1) [35,36,38–40]. With *h*SCAD, for example, the ideal ligand, butyryl-CoA, shifts the reduction potential positively, while longer acyl chains lead to a negative shift [40]. Similar down-regulating behavior observed in AidB with IVCoA could indicate that an increase of the AidB flavin reduction potential takes place upon binding to its as yet unknown biological substrate, and that once again IVCoA is not the relevant substrate.

The observed reduction potential modulations upon IVCoA binding are unusual because there is no obvious change in the FAD absorption spectrum upon IVCoA binding. Thus, the interactions between the acyl-CoA and AidB do not seem to have major structural impact on the FAD isoalloxazine ring. This finding is also consistent with semiquinone yield remaining constant. Unfortunately, we could not characterize the interaction between AidB and IVCoA using isothermal titration calorimetry due to the instability of AidB in the time course of the experiment. However, by taking advantage of the AidB FAD reduction potential shift upon IVCoA binding, we determined the apparent dissociation constant between AidB and IVCoA to be 87 μM. This binding affinity for IVCoA is, not surprisingly, weak compared to those of ACADs for their biologically relevant substrates (see for example references [40–44] and references therein). The reduced affinity for IVCoA, however, does not fully explain why the AidB dehydrogenase activity is so weak. Instead, our results suggest that unfavorable redox thermodynamics are the major factors limiting catalysis.

When DNA binds to AidB, we observed an up-shift of AidB flavin reduction potentials. This up-shift was observed with both plasmid and a double stranded 28-mer to which AidB preferentially binds *in vivo*[19], with the caveat that strong binding occurs at low ionic strengths which themselves have a drastic negative effect on reduction potential. The redox dependence on ionic strength marginalizes our observed regulation of reduction potential by DNA, as it is more likely that simple counter-ions are maintaining high reduction potentials *in vivo*. When DNA is present with 100 mM NaCl during *in vitro* experiments, some of these counter-ions are likely provided by the charged phosphate backbone, allowing for only a modest shift in reduction potential when compared to the effects of 300 mM NaCl. These data suggest that the presence of DNA does not have a strong effect on the flavin reactivity in AidB, a hypothesis that agrees with *in vivo* studies where the DNA-binding domain is removed without affecting AidB’s detoxification activity [19].

For AidB to catalyze a redox reaction, a mechanism would have to be in place to restore the oxidation state of the flavin cofactor, either reoxidizing reduced flavin after oxidation of a substrate by AidB, or *vice versa*. For ACADs, reoxidation of flavin occurs by two one-electron transfers to two ETFs [33,45]. ETFs are thought to form transient complexes with their different partner proteins, accepting electrons from different redox cofactors through reduction of their own flavin cofactor [25]. Efficient complex formation is dependent on docking of the recognition loop of the ETF to a hydrophobic pocket on the surface of its partner protein [28,29]. In our docking experiments, we find that this interaction is not conserved for AidB. Although modeling experiments cannot rule out an AidB-ETF interaction, the absence of the structural motifs in AidB thought to be crucial for this protein:protein interaction suggests that ETF is not a physiological electron acceptor for AidB. Thus, both the reductive and the oxidative half reactions of the AidB flavin appear to be different from the ACAD family. Since the chemistry of AidB has not been established, we do not know if flavin needs to be reoxidized, like ACADs by ETF as described above, or rereduced to catalyze its reaction. In this light, Mulrooney *et al*. investigated flavin reduction in AidB by flavodoxin (FldA) [20]. Flavodoxin is reported to bind to AidB and reduce flavin in AidB to the semiquinone state, but at a slow rate [20]. However, our electrochemical results demonstrate that the midpoint potentials of AidB (*E_m_^ox/sq^* = −103 mV, *E_m_^sq/red^* = −258 mV) and FldA*^ox/sq^* (–285 mV, [46]) are poised for effective electron transfer. Despite an appropriate reduction potential and binding affinity [20] the reduction of AidB by flavodoxin is sluggish, possibly due to the fact that the flavin in AidB is far from any accessible surface. The physiological relevance, if any, of this slow reduction by flavodoxin remains to be determined.

Overall, AidB exhibits major differences to its ACAD sequence homologs in terms of the redox chemistry of the associated flavin cofactor. These differences could be important for the divergent role of AidB. While ACADs perform oxidations of acyl chains, AidB has an important function in the bacterial adaptive response to alkylating agents. In particular, recent studies have demonstrated that AidB exerts a general protective effect on DNA against alkylation [19]. The protection itself does not rely on the DNA-binding domain, as demonstrated by studies with a truncated mutant lacking the DNA-binding domain. In agreement with these results, we do not observe a change in the AidB reduction potential in the presence or absence of DNA. The DNA-binding capacity of AidB instead appears to be important to localize AidB to so-called UP elements, resulting in increased protection of the crucial downstream genes such as ribosomal RNA genes, tRNA genes, and DNA repair genes [19].

Our electrochemical studies confirm that AidB is indeed poised for redox chemistry. The accumulated data point toward a function of AidB in the detoxification of alkylating agents, either by oxidation or by reduction, to prevent, rather than repair, DNA damage. Such a reaction could occur through one-electron chemistry that AidB can engage in, as indicated by the high degree of semiquinone stabilization. Possible substrates for AidB could be alkylating agents such as MNNG and MMS, although AidB was shown not to react with MNNG [20], or their metabolically activated forms, the exact natures of which are still unknown. Other hypotheses, such as direct DNA repair or a distinct ACAD activity, seem improbable at this point. The major task at hand now is the identification of AidB’s substrate, which will likely reveal the function of this enigmatic protein.

## 4. Experimental Section

### 4.1. Redox Potentiometry

Reduction potentials of the AidB FAD cofactor were measured using the xanthine/xanthine oxidase system described by Massey [47]. All measurements were conducted under anaerobic conditions (100% N_2_) in an MBraun glovebox using a S. I. Photonics 400 series spectrophotometer. *E. coli* AidB was prepared as described elsewhere [21]. The solution contained ~10 μM freshly purified protein in buffer (50 mM Tris, pH 8.0, 1 mM ethylenediaminetetraacetic acid, 300 mM NaCl, 10% (*v*/*v*) glycerol, 5 mM β-mercaptoethanol, 3–5 μM phenosafranine (PS), 1 μM benzyl viologen, 1 μM methyl viologen, and 350 μM xanthine). PS was added as the reference dye, and benzyl viologen and methyl viologen were included to ensure rapid equilibrium, but at small concentrations that would not perturb the spectra. Reduction of the mixture was initiated by the addition of 500 nM xanthine oxidase and full spectra were collected every 3–5 min until completion. When IVCoA or DNA was included, time was allowed for equilibration before the addition of xanthine oxidase. AidB absorbance was isosbestic at 555 nm so it could be used to monitor concentrations of oxidized and reduced PS. AidB reduction was monitored at the PS isosbestic point, 406 nm. Oxidized (ox) to reduced (red) absorbance transitions for PS and AidB were plotted as log ([ox]/[red])_PS_*vs*. log ([ox]/[red])_AidB_. From this plot, AidB reduction potentials (*E*_m_) were calculated based on the known reduction potential of PS, −283 mV at pH 8 [48]. All potentials reported are *versus* the standard hydrogen electrode.

The maximum amount of semiquinone intermediate (sq) formed during the experiment was determined as previously described [47]. The reduction of AidB was monitored at two wavelengths representing the oxidized (440 nm) and semiquinone (365 nm) forms. The plot of these two wavelengths during the course of reduction has two linear regions. The intercept of these linear fits is determined and the y-coordinate of this intercept corresponds to the theoretical absorbance of semiquinone at 365 nm if 100% semiquinone was formed. The experimental maximum semiquinone absorbance can then be used to determine the maximum proportion of semiquinone formed, ([sq]/[AidB])_max_.

### 4.2. Structural Modeling of Electron Transfer Flavoprotein Docking

To generate the docking model of AidB and human electron transfer flavoprotein (ETF), a dimer or tetramer of AidB (PDB ID: 3U33 [21]) was aligned with chain D of MCAD from the MCAD-ETF complex structure (PDB ID: 1T9G [29]) by a sequence-independent structure-based alignment in PyMOL [49]. Figures of the docking were generated in PyMOL [49].

## 5. Conclusions

We have characterized the redox properties of the AidB FAD cofactor, whose reduction potentials are well within the known flavin reduction potential range for biological chemistries. Like ACADs, the binding of CoA thioesters alters the redox properties of the flavin, but unlike ACADs, the semiquinone form of the flavin is highly stabilized. The reduction potentials of AidB are affected more dramatically by ionic strength than by the presence of DNA, suggesting that the adaptation of the ACAD-fold for DNA binding, which is observed in the AidB protein, is largely inconsequential in terms of redox chemistry. While flavodoxin can reduce AidB slowly, docking studies suggest that unlike members of the ACAD family, ETF would not be a good electron acceptor for AidB. Overall, while there are structural and redox similarities to the ACAD family of enzymes, AidB is not poised to carry out a reaction by the typical ACAD mechanism. Since the reduction potentials of AidB are in a biologically relevant range, our work is consistent with AidB being a redox active enzyme. However, the mechanism that AidB employs will likely have diverged from that engaged by ACAD family members.

## Figures and Tables

**Table 1:** Comparison of AidB with previously reported ACAD reduction potentials in mV. Measurements are listed for free enzymes as well as proteins in the presence of substrate (S), product (P), or non-optimal substrates described in the text or corresponding references.

Protein	*E_m_^ox/sq^*	*E_m_^sq/red^*	*E_m_^ox/red^*	% sq	pH	Radical	Ref.
AidB (300 mM NaCl)	−103	−258	−181	92	8.0	anionic	-
AidB + IVCoA (300 mM NaCl)	−148	−297	−222	91	8.0	anionic	-
AidB (100 mM NaCl)	−160	−315	−237	92	8.0	anionic	-
AidB + pUC19 (100 mM NaCl)	−121	−274	−197	92	8.0	anionic	-
AidB + 28-mer (100 mM NaCl)	−129	−282	−205	92	8.0	anionic	-

*Me*SCAD	-	-	−79	5	7.0	neutral	[35]
*Me*SCAD + S/P	-	-	−19	0	7.0	-	[37]
*Me*SCAD + Butyl-CoA	-	-	−79	0	6.9	-	[36]
*Me*SCAD + Acetoacetyl-CoA	-	-	−180	0	7.0	-	[35]

*Pd*GCD	-	-	−85	0	6.4	-	[39]
*Pd*GCD + S/P	-	-	30	5	6.4	anionic	[39]
*Pd*GCD + Acetoacetyl-CoA	−154 †	−104 †	−129	15	6.4	neutral	[39]

*p*MCAD	−166	−129	−136	20	7.6	neutral	[38]
*p*MCAD + S/P	-	-	−26	0	7.6	-	[34]
*p*MCAD + Butyl-CoA	−189 †	−155 †	−172	20	7.6	neutral	[38]

*h*SCAD *	-	-	−141	≤5	7.6	-	[40]
*h*SCAD * + Butyryl-CoA	-	-	−103	≤5	7.6	-	[40]
*h*SCAD * + Octanoyl-CoA	-	-	−161	≤5	7.6	-	[40]

* *h*SCAD reduction potentials reported for inactivated mutant enzyme.

† Values calculated from reported *E_m_* and percent semiquinone values.
